# Prognostic nomogram for patients with advanced unresectable hepatocellular carcinoma treated with TAE combined with HAIC

**DOI:** 10.3389/fphar.2024.1426912

**Published:** 2024-08-21

**Authors:** Li-xin Du, Guo-li Sheng, An-da Shi, Kang-shuai Li, Zeng-li Liu, Yong-chang Tang, Yi Liu, Zong-li Zhang

**Affiliations:** Department of General Surgery, Qilu Hospital, Cheeloo College of Medicine, Shandong University, Jinan, China

**Keywords:** unresectable hepatocellular carcinoma, HAIC, TAE, survival analysis, prognostic model

## Abstract

**Background:**

Hepatocellular carcinoma (HCC) is the most common primary liver cancer and often arises in the context of chronic liver disease, such as hepatitis B or C infection, and cirrhosis. Advanced unresectable HCC (uHCC) presents significant treatment challenges due to its advanced stage and inoperability. One efficient treatment method for advanced uHCC is the use of hepatic arterial infusion chemotherapy (HAIC) combined with transcatheter arterial embolization (TAE).

**Patients and Methods:**

In this study, we conducted a retrospective collection of clinical data, including basic information, radiological data, and blood test parameters, for patients with advanced uHCC who underwent TAE + HAIC treatment from August 2020 to February 2023. A total of 743 cases involving 262 patients were included. Ultimately, the covariates included in the analysis were the Child-Pugh score, extrahepatic metastasis, tumor number, tumor size, and treatment method.

**Results:**

In the study, we performed univariable and multivariable analysis on 23 clinical factors that were screened by LASSO regression, indicating that the five variables aforementionedly were identified as independent factors influencing patient prognosis. Then we developed a nomogram of the sensitive model and calculated concordance indices of prognostic survival models.

**Conclusion:**

Based on the uHCC patient cohort, we have developed a prognostic model for OS in patients who received TAE + HAIC treatment. This model can accurately predict OS and has the potential to assist in personalized clinical decision-making.

## Highlights

- The prognostic model for OS has been developed specifically for patients with advanced uHCC who have received TAE + HAIC.
- The model has the potential to assist in personalized clinical decision-making and prognosis assessment, providing a better net benefit than CNLC or BCLC tumor staging.

## Introduction

Malignant hepatic tumors refer to a group of liver cancers. According to the origin of the tumor, malignant hepatic tumors can be divided into primary cancers and metastatic tumors (Shi and Line, 2014; Sunnapwar et al., 2016; Esparza-Baquer et al., 2021). Hepatocellular carcinoma (HCC) is the most common primary malignancy of the liver (Perugorria et al., 2019; An et al., 2022). Advanced unresectable HCC (uHCC) was characterized by strong tumor invasiveness, limited treatment options, a very high patient mortality rate, and an inferior prognosis (Wang et al., 2022; Shi et al., 2024), and the 1-year survival rate for patients is approximately 20% (Gravitz, 2014). Patients with advanced uHCC cannot undergo radical resection treatment due to the challenging surgery and high mortality associated with the disease (Sangro et al., 2012; Galle et al., 2017), and one efficient treatment method for advanced uHCC is the use of TAE combined with HAIC (Chen et al., 2021; An et al., 2022).

TAE + HAIC is an interventional radiology procedure in which chemotherapy drugs and embolic agents are delivered directly into the main blood supply of the advanced uHCC, using a catheter. TAE + HAIC has shown promise in treating advanced uHCC, particularly in cases where the tumor is confined to the hepatic and has not spread extensively to other parts of the body (Lyu et al., 2022).

For advanced uHCC, radical surgical resection can not be performed to obtain visualized tumor data, and it is difficult to conduct predictive analysis based on the Barcelona Clinic Liver Cancer (BCLC) staging system (Chun et al., 2018; Banales et al., 2020). A novel prognostic assessment system needs to be established for predicting the prognosis of patients with advanced uHCC.

In this study, we developed a nomogram of the sensitive model and calculated concordance indices of prognostic survival models. This model, incorporating variables derived from univariable and multivariable regression analyses, can accurately predict the OS of patients with advanced uHCC who have undergone TAE + HAIC. The model has the potential to assist in personalized clinical decision-making and prognosis assessment.

## Materials and methods

### Patients

Clinical data of 262 patients with advanced uHCC who underwent combined therapy from 2020.08 to 2023.02 in Qilu Hospital of Shandong University, were retrospectively collected. HCC was diagnosed according to the guidelines of the American Association for the Study of Liver Diseases and the European Association for the Study of the Liver. The inclusion criteria were as follows: 1) age 18–80 years; 2) Child-Pugh class A or B liver function; 3) Eastern Cooperative Oncology Group performance score ≤1; 4) lack of preoperative adjuvant treatments for HCC; 5) no presence of other malignancies; 6) availability of complete clinical and follow-up data.

### Follow-up

To ensure the accuracy of survival data, follow-up assessments of patients were scheduled every 3 months. All patients were reviewed at the last follow-up in June 2023. Overall survival (OS) was defined as the duration from the initial treatment to either the patient’s death or the last follow-up.

### Cohort definition

Patients were randomly divided into training and validation cohorts in a 7:3 ratio to ensure an even distribution of outcome events. The demographic characteristics, laboratory data, and radiological data of patients in the two cohorts were comparable. The training cohort was used to screen variables and construct the nomogram, while the validation cohort was used to validate the results.

### Variable selection

As described herein, a total of 23 variables were included in the selection process. To minimize the potential collinearity and over-fitting of variables, the Least Absolute Shrinkage and Selection Operator (LASSO) regression was applied. The variables identified by LASSO regression were then entered into Univariate Cox regression analysis. Relevant variables with a *p*-value of less than 0.10 were selected as inputs for further analysis. Forward-backward stepwise selection based on the Akaike information criterion (AIC) was used to evaluate variables for the multivariate COX regression model (Collins et al., 2015). Hazard ratios and their corresponding 95% confidence intervals (CIs) were calculated.

### Development and validation of nomogram

Significant variables were incorporated into a nomogram to predict the 6-, 12-, and 18-month OS rates after initial treatment. Calibration curves were utilized to assess the agreement between predicted survival probabilities and actual survival proportions at each time point (6, 12, 18 months). Discriminative ability was evaluated using the Concordance index (C-index) and area under the time-dependent receiver operating characteristic curve (time-dependent ROC). Additionally, decision curve analysis was conducted to determine the clinical utility of the nomogram by quantifying the net benefits at different threshold probabilities compared with CNLC or BCLC tumor staging (Vickers et al., 2008).

### Statistical analysis

All statistical analysis was conducted using R version 4.3.1 (R Foundation for Statistical Computing). Continuous variables are presented as the mean ± standard deviation or the median with interquartile range (IQR). Categorical variables were presented as frequencies and proportions. Survival curves were generated using the Kaplan-Meier method, and the log-rank test was employed to compare curves. A two-tailed *p*-value of less than 0.05 was considered statistically significant.

## Results

### Baseline characteristics of the cohort

**FIGURE 1:**
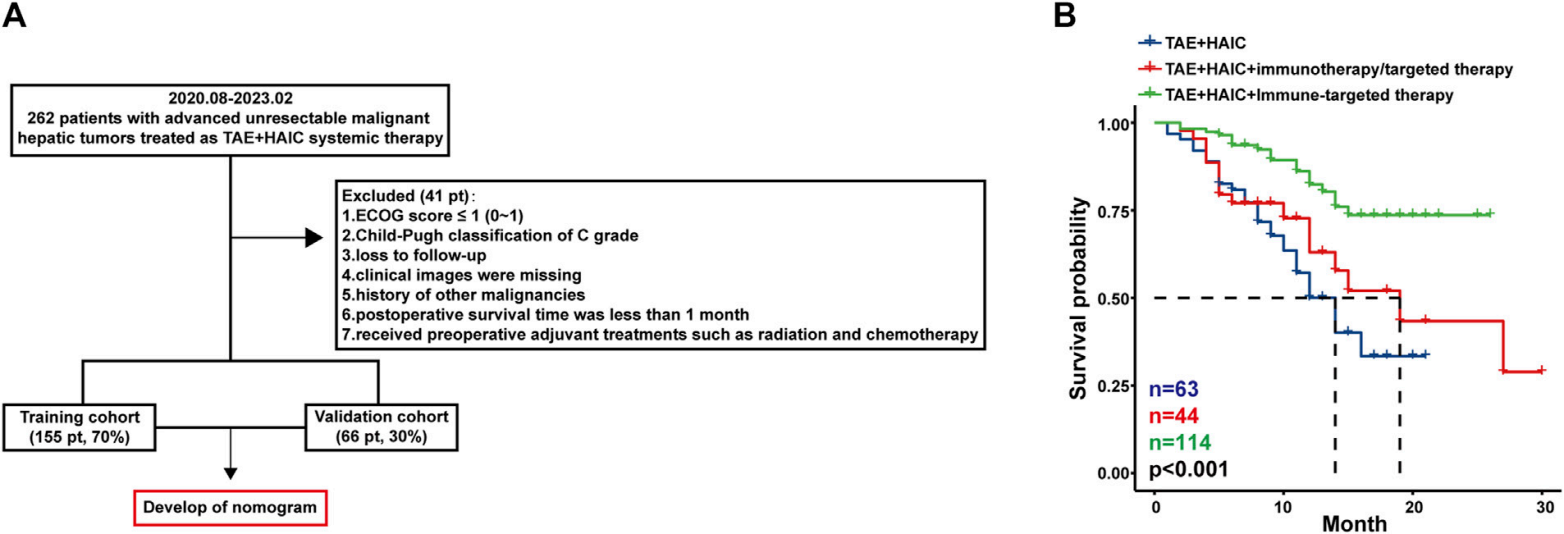
The patient flow of the study. Pt, patients. Flow chart **(A)** and survival curves **(B)** of the cohort in patients with advanced uHCC.

A total of 743 cases involving 262 patients with advanced uHCC who have undergone TAE + HAIC were included in this analysis, and 41 patients were excluded primarily due to loss of follow-up and missing clinical data, of which 23 (8.8%) were lost of follow-up. The patients were divided into the training and validation cohorts with a ratio of 7:3 to ensure that outcome events were distributed randomly between the two cohorts (Figure 1A). In an overall perspective, The median overall survival (OS) for 63 patients treated with TAE + HAIC was 14.0 months (95% CI: 11.4–16.2). For 44 patients treated with TAE + HAIC followed by immunotherapy or targeted therapy, the median OS was 19.0 months (95% CI: 11.1–26.9). For 114 patients treated with TAE + HAIC followed by Immune-targeted therapy, the OS rate exceeded 50% at 30 months (Figure 1B). The baseline clinicopathological characteristics of advanced uHCC were obtained from medical records. These clinical factors will be further screened through LASSO regression and then included in Cox regression as foundational factors for developing the nomogram model (Table 1).

**TABLE 1:** Clinicopathological characteristics of patients with advanced unresectable hepatocellular carcinoma who underwent TAE + HAIC.

Characteristic	Total	Training cohort	Validation cohort	*p*-value
	N = 221	N = 155	N = 66	
Gender				0.55
Female	56 (25.3%)	37 (23.9%)	19 (28.8%)	
Male	165 (74.7%)	118 (76.1%)	47 (71.2%)	
Age, years	59.0 (52.0, 65.0)	58.0 (52.0, 66.0)	59.0 (52.0, 64.8)	0.78
HAIC session	3.00 (2.00, 4.00)	3.00 (2.00, 4.00)	3.00 (1.25, 4.00)	0.78
Therapy				1.00
TAE + HAIC	63 (28.5%)	44 (28.4%)	19 (28.8%)	
TAE + HAIC + immunotherapy/targeted therapy	44 (19.9%)	31 (20.0%)	13 (19.7%)	
TAE + HAIC + Immune-targeted therapy	114 (51.6%)	80 (51.6%)	34 (51.5%)	
Etiology				0.38
Nonhepatitis	133 (60.2%)	89 (57.4%)	44 (66.7%)	
HCV	2 (0.90%)	2 (1.29%)	0 (0.00%)	
HBV	86 (38.9%)	64 (41.3%)	22 (33.3%)	
Tumor size (cm)				0.63
≤5	67 (30.3%)	45 (29.0%)	22 (33.3%)	
>5	154 (69.7%)	110 (71.0%)	44 (66.7%)	
Tumor number				0.06
<3	120 (54.3%)	91 (58.7%)	29 (43.9%)	
≥3	101 (45.7%)	64 (41.3%)	37 (56.1%)	
Liver lobes invasion				0.33
Single	120 (54.3%)	88 (56.8%)	32 (48.5%)	
Double	101 (45.7%)	67 (43.2%)	34 (51.5%)	
Vascular invasion				0.47
Absence	150 (67.9%)	108 (69.7%)	42 (63.6%)	
Presence	71 (32.1%)	47 (30.3%)	24 (36.4%)	
Portal vein tumor thrombus				0.73
Vp0	150 (67.9%)	108 (69.7%)	42 (63.6%)	
Vp1-2	6 (2.71%)	4 (2.58%)	2 (3.03%)	
Vp3	24 (10.9%)	17 (11.0%)	7 (10.6%)	
Vp4	41 (18.6%)	26 (16.8%)	15 (22.7%)	
Extrahepatic metastasis				0.16
Absence	204 (92.3%)	140 (90.3%)	64 (97.0%)	
Presence	17 (7.69%)	15 (9.68%)	2 (3.03%)	
Child-Pugh score				0.65
A	199 (90.0%)	141 (91.0%)	58 (87.9%)	
B	22 (9.95%)	14 (9.03%)	8 (12.1%)	
ECOG				0.29
0	191 (86.4%)	131 (84.5%)	60 (90.9%)	
1	30 (13.6%)	24 (15.5%)	6 (9.09%)	
CNLC stage				0.80
I	76 (34.4%)	55 (35.5%)	21 (31.8%)	
II	68 (30.8%)	48 (31.0%)	20 (30.3%)	
III	77 (34.8%)	52 (33.5%)	25 (37.9%)	
BCLC stage				0.72
A	53 (24.0%)	39 (25.2%)	14 (21.2%)	
B	38 (17.2%)	25 (16.1%)	13 (19.7%)	
C	130 (58.8%)	91 (58.7%)	39 (59.1%)	
AFP, ng/mL				0.71
≤400	133 (60.2%)	95 (61.3%)	38 (57.6%)	
>400	88 (39.8%)	60 (38.7%)	28 (42.4%)	
WBC, ×109/L	5.14 (3.87, 6.65)	4.97 (3.94, 6.42)	5.54 (3.87, 6.69)	0.24
HGB, g/L	131.5 ± 18.4	130.7 ± 18.0	133.5 ± 19.1	0.31
PLT, ×109/L	181 (132, 234)	178 (132, 239)	188 (130, 226)	0.88
ALT, U/L	33.0 (19.0, 52.0)	34.0 (20.0, 53.0)	32.0 (18.2, 51.2)	0.95
GGT, U/L	127 (63.0, 215)	133 (65.0, 216)	109 (61.2, 205)	0.56
AKP, U/L	128 (99.0, 167)	130 (98.5, 166)	122 (104, 167)	0.99
PT, s	12.1 (11.3, 13.0)	12.1 (11.3, 12.9)	12.1 (11.3, 13.1)	0.90

Abbreviations: HAIC, hepatic arterial infusion chemotherapy; TAE, transcatheter arterial embolization intervention.

### Regression analysis and nomogram construction in the advanced uHCC cohort

**FIGURE 2:**
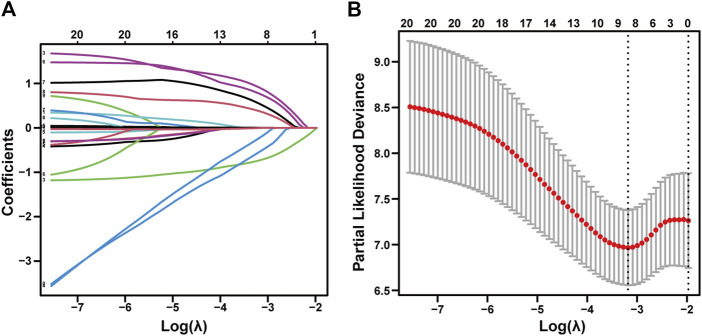
LASSO regression of advanced uHCC cohort. Feature selection using the least absolute shrinkage and selection operator. **(A)** LASSO coefficient profiles of the 23 baseline features. **(B)** The selection process of the optimum value of the parameter λ in the Lasso regression model by a 10-fold cross-validation method.

As described herein, we performed LASSO regression to screen the collected 23 variables and calculated the regression coefficients for each clinical factor (Figure 2A). Nine resolving variables were selected for further analysis, including Child-Pugh score, extrahepatic metastasis, tumor number, tumor size, and treatment method, etiology, portal vein tumor thrombus, age, HAIC session (Figure 2B). After conducting univariable and multivariable analysis on the selected nine clinical factors, we included variables that exhibited a significant association with OS to identify independent predictors of OS (Table 2).

**TABLE 2:** Cox regression analysis of predictive factors for overall survival in patients with advanced unresectable hepatocellular carcinoma.

	Univariable analysis		Multivariable analysis	
Characteristics	HR (95% CI)	*p*-value	HR (95% CI)	*p*-value
Therapy (TAE + HAIC + immunotherapy/targeted therapy vs. TAE + HAIC)	0.80 (0.38–1.67)	0.546	0.76 (0.36–1.59)	0.465
Therapy (TAE + HAIC + Immune-targeted therapy vs. TAE + HAIC)	0.33 (0.17–0.66)	0.002	0.27 (0.14–0.55)	<0.001
Age	1.03 (1.00–1.06)	0.083		
Tumor size	1.97 (0.95–4.09)	0.068	3.04 (1.43–6.47)	0.004
Tumor number	1.75 (0.98–3.13)	0.058	2.29 (1.26–4.17)	0.007
Extrahepatic metastasis	2.45 (1.03–5.82)	0.042	3.73 (1.75–7.96)	<0.001
Child-pugh (B vs. A)	2.60 (1.21–5.61)	0.015	3.67 (1.65–8.17)	<0.001
HAIC session	0.87 (0.73–1.03)	0.102		

Abbreviations: HR, hazard ratio; CI, confidence interval.

^a^ Calculated by log-rank test.

^b^ Calculated by Cox-regression Hazard model.

**FIGURE 3:**
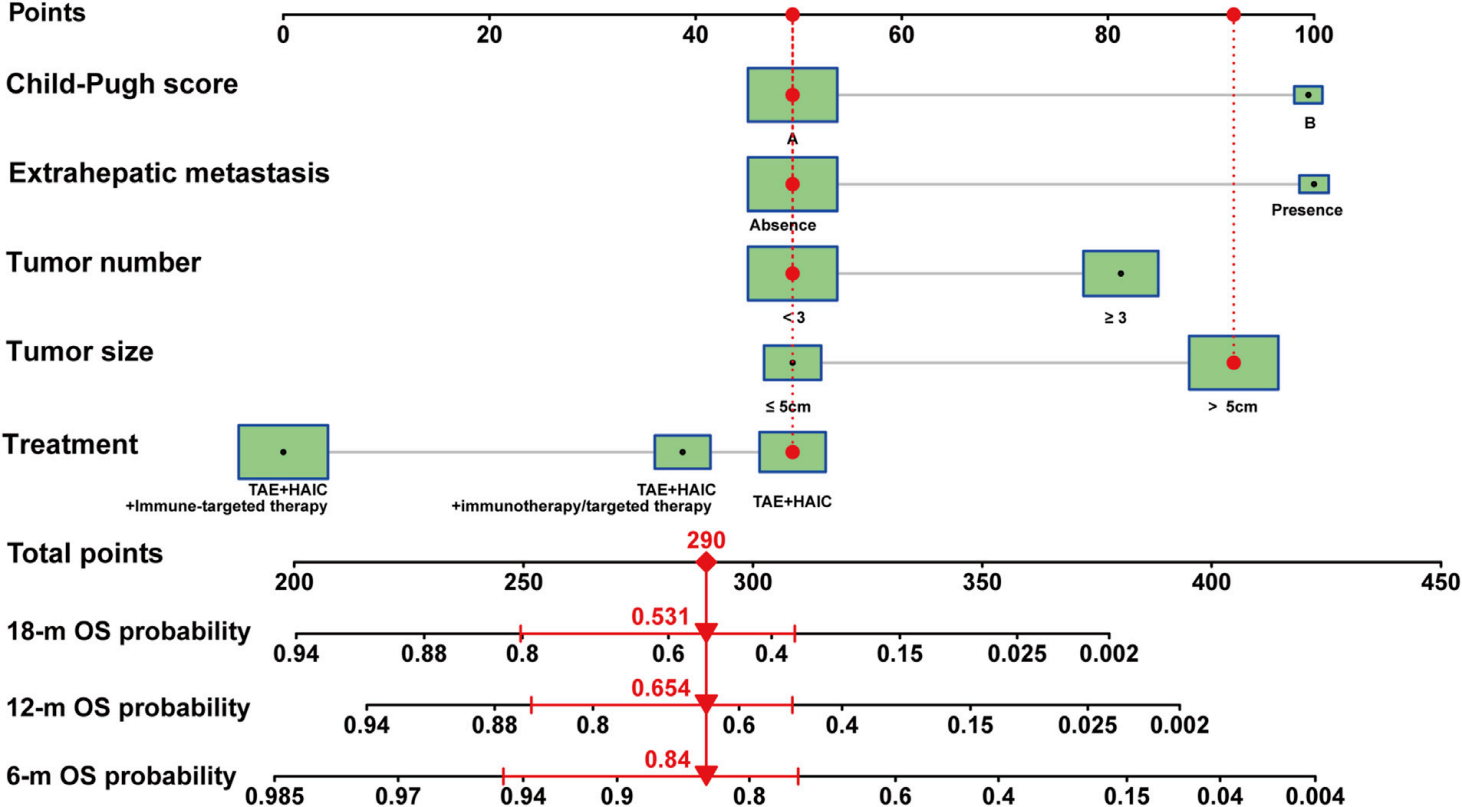
Nomogram model of advanced uHCC cohort. Nomogram for prognostic prediction of a patient with advanced uHCC. The patient had a single, local tumor of 6.1 cm, with liver function is Child-pugh A, without extrahepatic metastasis, and underwent HAIC therapy. For category variables, their distributions are reflected by the size of the box. The importance of each variable was ranked according to the standard deviation along nomogram scales. To use the nomogram, the specific points (black dots) of individual patients are located on each variable axis. Red lines and dots are drawn upward to determine the points received by each variable; the sum (290) of these points is located on the Total Points axis, and a line is drawn downward to the survival axes to determine the probability of 6- month (84.0%), 12-month (65.4%) and 18-month (53.1%) overall survival.

Subsequently, we developed nomograms using the outcomes of the multivariable analysis, aiming to forecast 6-month, 12-month, and 18-month OS after interventional surgery involving HAIC plus TAE for advanced uHCC (Figure 3). A higher total score was correlated with poorer OS, and the nomogram demonstrated a concordance index (C-index) of 0.74 (training cohort, 95% CI: 0.66–0.82) and 0.76 (validation cohort, 95% CI: 0.64–0.89) for OS prediction.

### Performance analysis of nomogram

**FIGURE 4:**
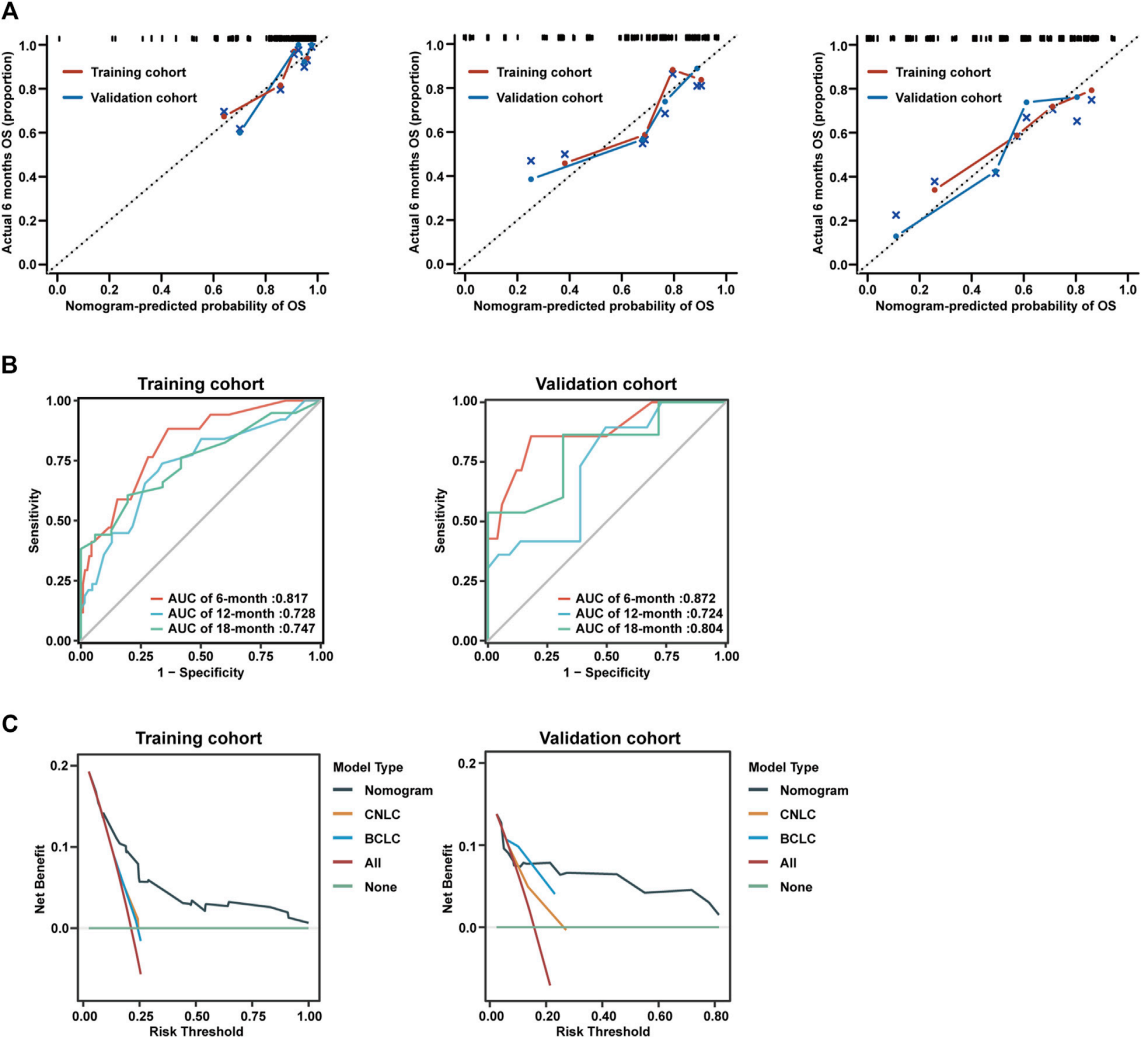
Calibration curves and decision curve analysis of nomogram model. **(A)** Calibration curves of 6-month (left), 12-month (middle), and 18-month (right) OS for uHCC patients in the training cohort and validation cohort. **(B)** AUC of using the nomogram to predict overall survival probability in the training cohort and validation cohorts. **(C)** Decision curve analysis of the nomogram, CNLC tumor staging, and BCLC tumor staging for the survival prediction of patients with advanced uHCC.

To assess the performance of the nomogram model, calibration curves for 6-month, 12-month, and 18-month were generated, which showed strong concordance between the predicted and observed probabilities of OS (Figure 4A). In addition, AUC curves for training and validation cohorts were generated for OS status, considering cumulative sensitivity (Figure 4B).

To evaluate the prognostic performance and clinical utility, we conducted a comparative analysis of the nomogram model and CNLC or BCLC tumor staging using Decision Curve Analysis (DCA). The findings indicated that the nomogram model consistently provided a superior net benefit compared to CNLC or BCLC tumor staging across various thresholds for advanced uHCC (Figure 4C).

## Discussion

Advanced uHCC exhibited high tumor heterogeneity, poor prognosis, and an exceedingly high treatment risk (Wang et al., 2022). As the primary treatment approach for advanced uHCC (Zhou and Song, 2021), the effectiveness and postoperative prognosis prediction model of interventional therapy should be studied.

Advanced uHCC typically has a plentiful blood supply, but embolization can help reduce the supply to the tumor. White ball embolization can also increase the duration that chemotherapy drugs interact with the tumor, ultimately improving their effectiveness. While there was a consensus that TAE + HAIC provided benefits to patients with advanced uHCC (Forner et al., 2018; Yang et al., 2023), there has been no research on the construction of a predictive model for the postoperative prognosis of TAE + HAIC-treated patients. In consideration of this circumstance, we conducted a retrospective data collection and established a cohort comprising patients with advanced uHCC who underwent TAE + HAIC intervention surgery, subsequently tracking their survival data. Through LASSO regression screening and logistic regression analysis of the gathered clinical data, we once again validated that specific clinical factors act as independent risk factors for patient prognosis.

Accurate prognostic models are crucial for forecasting patient outcomes, guiding adjuvant therapy, and informing postoperative monitoring in cancer patients (Groot Koerkamp et al., 2015; Burkhart and Pawlik, 2017). At present, there exists no established effective model for predicting the survival situation of post-TAE + HAIC patients (Chen et al., 2021). Therefore, we have developed a nomogram of the sensitive model to anticipate the expected survival outcomes for post-TAE + HAIC patients. Advanced uHCC patients cannot undergo radical resection treatment (Sangro et al., 2012; Galle et al., 2017), it is therefore hard to obtain pathological data and conduct predictive analysis based on the BCLC staging system (Anwanwan et al., 2020). To the best of our abilities, we gathered general clinical information and various clinical data from malignant uHCC patients and predicted variables that could impact the outcomes. We conducted stepwise regression analysis on these variables and ultimately chose the essential clinical factors for nomogram model construction. This improved the model’s sensitivity, accuracy, and stability in predicting post-TAE + HAIC patient survival rates, consequently guiding clinical treatment. The data analysis, which included assessing the C-index and calibration plots, confirmed that the nomogram demonstrated a high level of prediction accuracy. Furthermore, we observed a high level of accuracy in predicting OS using this nomogram. The nomogram model exhibited a superior net benefit to CNLC or BCLC tumor staging following the TAE + HAIC intervention. This discovery suggests that the nomogram can serve as a valuable addition to clinical practice, enabling prognosis assessment for patients with advanced uHCC.

Our study has several noteworthy advantages. Firstly, the sample cohort of advanced uHCC patients is relatively large. Before excluding patients, a total of 743 cases involving 262 patients were included. This significant sample size enhances the confidence and precision of the predicted OS. Secondly, we established comprehensive clinical information, such as gender, age, history of hepatitis B, maximum tumor diameter, number of intrahepatic tumors, tumor distribution differentiation, the situation of invasion and metastasis, ascites, WBC, HGB, PLT, ALB, TBIL, DBIL, ALT, GGT, AKP, PT, and AFP, and accurate follow-up data for the included patients. The selection of multiple variables enhanced the scientific rigor of nomogram construction, making the research results more representative and reliable. Certainly, this study also exhibits several weaknesses. Firstly, this study is a single-center retrospective study, which may introduce potential selection bias. Secondly, further confirmation of the results’ applicability is required through large-scale prospective studies conducted across multiple centers, and the sample size of the uHCC cohort should be further expanded. In summary, although this study has a retrospective design, it provides valuable insights that can enhance the management of patients with advanced uHCC following TAE + HAIC and promote personalized treatment.

## Data Availability

The datasets presented in this article are not readily available due to privacy reasons. Requests to access the datasets should be directed to Z-lZ, zzlzzl1900@163.com.

## Abbreviations

BCLC, Barcelona Clinic Liver Cancer; CI, confidence interval; C-index, concordance index; DCA, decision curve analysisu; ECOG, Eastern Cooperative oncology Group; HCC, unresectable hepatocellular carcinoma; HAIC, hepatic arterial infusion chemotherapy; LASSO, least absolute shrinkage and selection operator; OS, overall survival; TAE, transcatheter arterial embolization intervention; TNM, tumor-node-metastasis; HR, hazard ratio.
